# The dual role of IL-17 in periodontitis regulating immunity and bone homeostasis

**DOI:** 10.3389/fimmu.2025.1578635

**Published:** 2025-04-03

**Authors:** Zhongyuan Tang, Lijian Jin, Yanqi Yang

**Affiliations:** ^1^ Division of Paediatric Dentistry and Orthodontics, Faculty of Dentistry, The University of Hong Kong, Hong Kong, Hong Kong SAR, China; ^2^ Division of Periodontology and Implant Dentistry, Faculty of Dentistry, The University of Hong Kong, Hong Kong, Hong Kong SAR, China

**Keywords:** IL-17, periodontitis, inflammation, bone, neutrophil

## Abstract

Periodontitis is a common dysbiotic bacteria-induced inflammatory disease characterized by alveolar bone resorption, leading to tooth loss. Interleukin-17 (IL-17) is a critical cytokine with dual roles in periodontium, which exerts the function of host defense, including neutrophil recruitment, phagocytosis, and mucosal immunity. However, excessive expression of IL-17 causes persistent chronic inflammation, local tissue breakdown, and bone loss. This review highlights the protective and pathological functions of IL-17 on immunity and bone homeostasis in inflammatory bone-related diseases. We also provide the latest findings with IL-17 knockout mice in periodontitis and highlight complex immune responses under various experimental models. This may provide a critical perception of inflammatory bone-related disease management using an immune-modulating strategy.

## 1 Introduction

Periodontitis, a widely prevalent inflammatory diseases, significantly impacts individual oral health. As a chronic inflammatory condition induced by bacterial dysbiosis, periodontitis compromises gingival health, destroys tooth-supporting tissue, such as alveolar bone and periodontal ligament, and causes tooth loss (1, 2). The dysbiosis in the periodontal bacterial community initiates periodontitis, activating a cascade of immune mediators that can be affected by external and systemic factors such as smoking, psychological stress, diabetes, and obesity (3–7). It is important to recognize that the etiology of periodontitis involves both inflammatory stimuli and the host immune response.

The immune response is essential for controlling infections when bacteria invade, but it can also exacerbate tissue destruction in chronic inflammation and autoimmune diseases (8). Among the immune cytokines, interleukin-17 (IL-17) has prominent functions in both defending against microbial infections and promoting pathological inflammation. IL-17 is produced by various immune cells, including Th17 cells, innate lymphoid cells, γδ T cells, and natural killer T cells, depending on the inflammatory context (9). However, IL-17 is primarily produced by Th17 cells and enhances neutrophil recruitment and mucosal immunity (10–13). However, excessive release of IL-17 is implicated in chronic inflammation, tissue breakdown, and bone loss, making it a critical focus of research in periodontitis and other bone-related inflammatory diseases (14, 15).

While current therapeutic strategies for periodontitis primarily focus on eliminating bacterial stimuli, there is a growing interest in modulating the host immune response to achieve better treatment outcomes (16). Targeting IL-17 offers a promising avenue for reducing its pathological effects on alveolar bone resorption without affecting its protective functions against bacterial pathogens in periodontitis. Although numerous studies have shown that excessive IL-17 can lead to chronic inflammation, directly contributing to increased bone resorption in inflammatory bone diseases such as autoimmune osteoarthritis, its role in bone-related diseases induced by infections or other complex factors remains controversial. This review aims to provide a comprehensive overview of studies using gene knockout mouse models to investigate IL-17’s roles in periodontal and other bone-related inflammatory diseases, including the protective and pathological functions, its impact on bone homeostasis, and its relevance in *Porphyromonas gingivalis* (*P. gingivalis*)-induced periodontitis. By addressing the controversies and limitations in current research, this review is expected to offer valuable insights for a deeper understanding of therapeutic strategies and mechanistic explorations, ultimately benefiting periodontitis and other bone-related inflammatory diseases in the future.

## 2 Microbial threats and immune responses in periodontitis

### 2.1 Microbial dysbiosis as a trigger

The progression of chronic periodontitis involves broader ecological shifts in the subgingival microbiota composition, rather than being driven by a single pathogen alone (17–19). Although the “red complex” species *Treponema denticola*, *Tannerella forsythia*, and particularly *P. gingivalis* (20) are frequently enriched in periodontitis lesions, current insights underscore that these bacteria orchestrate disease in the context of polymicrobial synergy and dysbiosis (17). For instance, *P. gingivalis* is a key immune regulator due to its unique pathogenicity, instead of acting as a direct inflammatory trigger (21, 22). By disrupting host immunity by interfering with Toll-like receptors (TLRs) and complement interactions (22, 23), *P. gingivalis* drives a microbial dysbiosis and unregulated inflammation, thereby fueling bone loss (24).

This dysbiosis is maintained through intricate interbacterial signaling and metabolic support among complementary community members that can flourish under inflammatory or nutrient-altered conditions (18). Emerging evidence shows *P. gingivalis* manipulates T-cell responses and promotes Th17-driven inflammation while suppressing Th1-mediated immunity (25), thus hindering effective bacterial clearance (26) and prolonging the inflammatory state (17). This immune modulation facilitates its survival and further triggers inflammatory bone loss (22). Additionally, commensal microorganisms, under disrupted homeostasis, can contribute to inflammation by activating pattern-recognition receptors (27). This interaction creates a nutrient-rich environment that benefits proteolytic and anaerobic species (28), which in turn exacerbates tissue damage and microbial proliferation. Consequently, a feed-forward loop of polymicrobial synergy perpetuates periodontal disease (17).

### 2.2 Host-driven immune responses

Periodontal tissue destruction is influenced not only by microbial agents but also by host susceptibility, which can be affected by immunodeficiencies, systemic conditions (e.g., diabetes, aging, obesity), stress, and dietary patterns (29–33). These factors disrupt host-microbe equilibrium and contribute to tissue homeostasis breakdown (34, 35). While dental plaque is a key factor in gingivitis—a reversible gingival inflammation without bone damage—it does not inherently lead to periodontitis. Some individuals maintain periodontal health despite significant plaque accumulation, reflecting differences in host resilience and genetic factors (29, 31). However, gingivitis can progress to periodontitis, where the inflammatory environment shifts to a state favoring opportunistic pathogens and a profound immune dysregulation.

During gingivitis, subgingival biofilms become enriched in Gram-negative and anaerobic bacteria compared to health, triggering localized recruitment of neutrophils, macrophages, and lymphocytes (36). These cells cause tissue-level inflammation yet often remain confined to the soft tissues. By contrast, in severe or chronic periodontitis, the dysbiotic microbiome, dominated by bacterial species better equipped to evade host immunity, fuels an intensified host response, leading to collagen breakdown and alveolar bone resorption (17, 36).

The immune system in the gingiva is particularly susceptible to microbial invasion due to the porous structure of the epithelium. The junctional epithelium, which provides a direct interface with the subgingival environment, is interlinked by gap junctions and desmosomes, offering limited physical barriers to microorganism infiltration (37). This structural vulnerability necessitates a robust immune response to continually counteract the colonization of periodontal pathogens. Immune cells such as neutrophils, macrophages, and lymphocytes are rapidly recruited to the site of bacteria accumulation, releasing various pro-inflammatory cytokines in the periodontium (38). Additionally, immune mediators such as TLRs, E-selectin, lipopolysaccharide-binding protein, intercellular adhesion molecules, IL-8, β-defensins, and soluble CD14 play a pivotal role in mobilizing leukocytes and controlling bacterial growth (39–45). These complex responses from host-microbe interactions determine whether early gingivitis resolves, remains stable, or advances to destructive periodontitis.

## 3 IL-17 family and IL-17-producing cells

IL-17, discovered several decades ago, is a cytokine essential for protecting mucosal barriers against microbial invasion and infection. However, it also plays a crucial role in autoimmune diseases and pathological inflammation during infection. The IL-17 family consists of six subtypes (IL-17A–F) that function through specific receptors (IL-17RA–E), each mediating distinct biological effects (46). Among these, IL-17A is the most extensively studied member and the first to be identified (47). IL-17A, along with IL-17F, plays a pivotal role in defending against bacterial and fungal infections, particularly in conditions such as periodontal disease. These cytokines share over 50% sequence identity across humans and mice, with core functions conserved between species (48), despite greater sequence divergence among other IL-17 family members (49). Compared to IL-17F, IL-17A has a high binding affinity for IL-17RA and IL-17RC, triggering immune responses that, while protective, may also lead to tissue damage (50). Additionally, some research has suggested that IL-17RA can be released in a soluble form, which may act as a potential inhibitor of IL-17A signaling (51, 52).

CD4+ T helper cells are a primary source of IL-17 (53). Th17 cells play a key role in recruiting neutrophils and enhancing innate immunity against extracellular microorganisms (54). Their differentiation is regulated by IL-6, transforming growth factor (TGF)-β, IL-1, and IL-21, with IL-23 essential for their growth and survival (55). RORγt is the master transcription factor that governs Th17 differentiation and activation (56). Although IL-17 was initially attributed primarily to Th17 cells in adaptive immunity, subsequent research has revealed that various innate immune cells also produce IL-17. These cells include γδ T cells, lymphoid tissue inducer cells, natural killer cells, Foxp3+ Treg-like cells, invariant natural killer T cells, T-cell receptor (TCR)αβ+ natural Th17 cells, and Paneth cells (9). Unlike CD4+ Th17 cells, these cellular populations secrete IL-17 without TCR activation. Instead, they generate IL-17 in response to specific factors such as TGF-β, IL-23, and IL-1β (57). Additionally, some evidence suggests that myeloid cells and B cells may express IL-17, although this remains controversial (58, 59). In healthy periodontal tissues, IL-17-producing cells are relatively rare. In humans, Th17 cells are the major source of IL-17 in gingiva. In contrast, due to limited pathogen exposure in laboratory mice, the proportion of adaptive T cells is relatively small, so innate T cells predominate (60). Under these conditions, γδ T cells are the primary producers of IL-17, followed by Th17 cells and a small subset of group 3 innate lymphoid cells (ILC3s) (61–63).

## 4 Immunoprotective roles of IL-17

**Figure 1:**
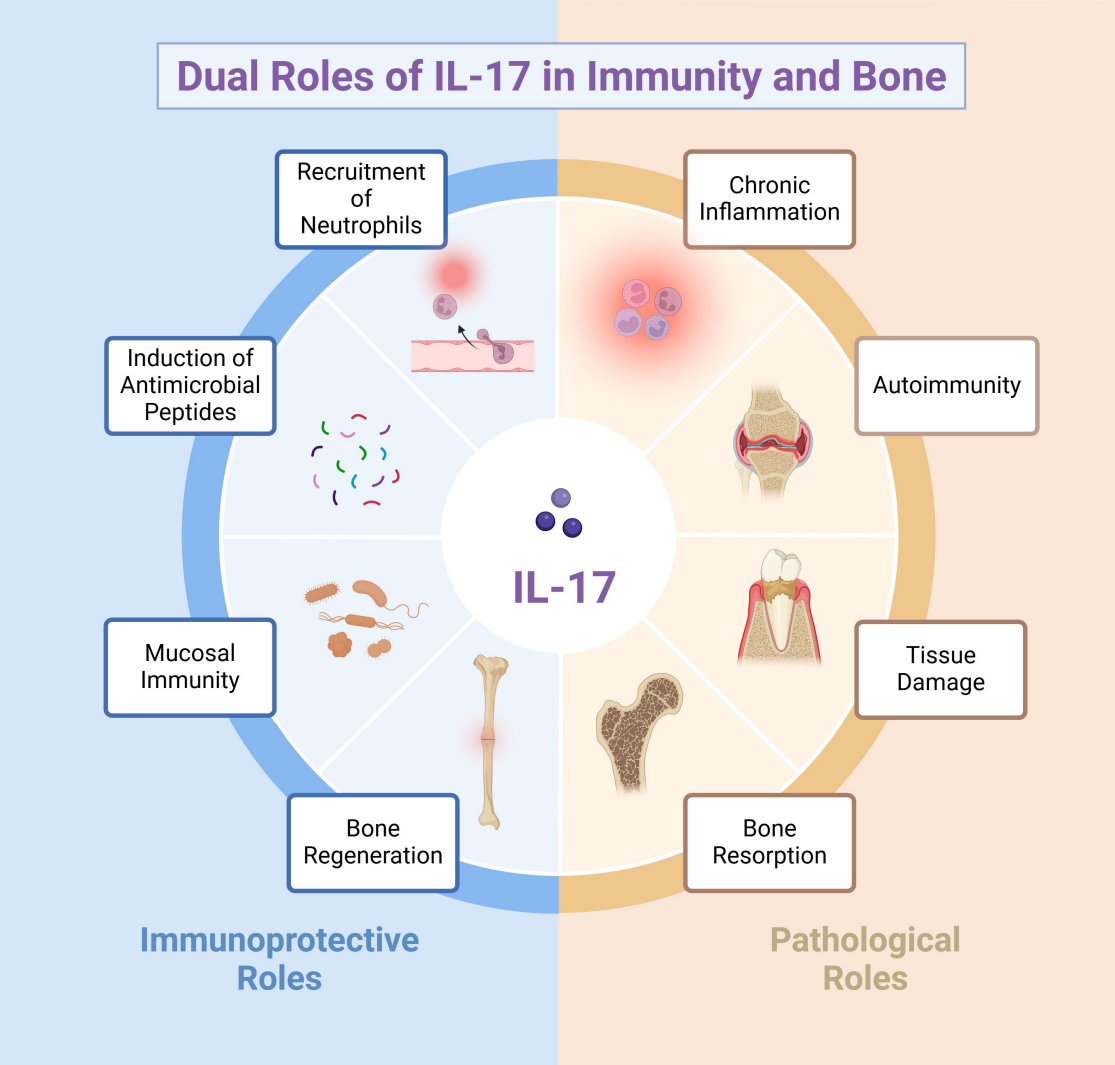
Schematic representation of the dual roles of IL-17 in immunity and bone. The left (blue) segment shows IL-17’s immunoprotective functions, including the recruitment of neutrophils, induction of antimicrobial peptides, support of mucosal immunity, and promotion of bone regeneration. Conversely, the right (peach) segment illustrates its pathological role in chronic inflammation, autoimmunity, tissue damage, and bone resorption. These contrasting effects underscore IL-17’s pivotal but complex influence in disease pathogenesis and bone homeostasis.

IL-17 is pivotal for orchestrating host defense against bacterial infections (**Figure 1**). One of its hallmark functions involves enhancing the chemotaxis of neutrophils to eliminate invading bacteria. The recruitment of polymorphonuclear neutrophils (PMNs) is mediated by IL-17-induced upregulation of chemokines, such as CXC chemokine ligands (CXCL) 1, CXCL2 and granulocyte colony-stimulating factor (G-CSF) (64). As a result, PMNs are mobilized from the bone marrow into the bloodstream and subsequently recruited to infected tissues. PMNs serve as a critical component of the innate immune response by phagocytosing pathogens, producing antimicrobial peptides (AMPs) (64), and releasing neutrophil extracellular traps (NETs) (65–67).

In models of *Klebsiella pneumoniae* (*K. pneumoniae*) infection in the intranasal mucosa, IL-17RA knockout mice exhibit significantly reduced expression of G-CSF and MIP2, as well as impaired neutrophil recruitment, leading to aggravated infection and even death. In the Helicobacter pylori-colonized gastric mucosa, IL-17 markedly inhibits IL-8 expression in gastric lamina propria mononuclear cells, thereby suppressing the recruitment of PMNs. In *P. gingivalis*-induced periodontitis, mice deficient in IL-17RA show impaired neutrophil recruitment, leading to higher microbial loads (68, 69), resulting in severe bone resorption (68).

IL-17 directly enhances the phagocytic activities of neutrophils and macrophages, thereby promoting bacterial killing and clearance. During lung infections, IL-17 can reduce the burdens of *Bordetella pertussis* (*B. pertussis*) (70), *Streptococcus pneumoniae* (*S. pneumoniae*) (71) and *Haemophilus influenzae* (72) by increasing neutrophil recruitment. In IL-17A knockout mice with *K. pneumoniae*-colonized pneumonia, impaired neutrophil recruitment and increased bacterial loads were observed (64).

IL-17 also maintains mucosal barrier integrity in multiple ways (64, 73). In IL-17A knockout mice with *B. pertussis*-colonized nasal mucosae, decreased AMPs production and weakened NETs activity were observed, leading to reduced bacterial clearance (73). The expansion of IL-17-secreting CD4^+^ tissue-resident memory T cells protects against respiratory pathogens such as *B. pertussis* and *S. pneumoniae* during acquired immunity by inducing the secretion of specific IgA and IgG in the nasal mucosa (73, 74). These T cells can further support neutrophil recruitment and defensin production (73). Studies have shown that vaccines protect against respiratory pathogens by stimulating IL-17 secretion from tissue-resident memory T cells (74–76).

Despite IL-17’s prominent role in neutrophil recruitment and chemotaxis, activation of phagocytic function, and promotion of mucosal immunity, its overall impact on tissue and host defense is highly context-dependent. In the microenvironments of most bacterial infectious diseases, IL-17–driven chemokine production and G-CSF release effectively mobilize neutrophils from the bone marrow and direct them to sites of infection, thereby facilitating pathogen clearance. In specific bacterial settings and disease models involving *B. pertussis* (70), *S. pneumoniae* (71) and *Haemophilus influenzae* (72), IL-17 further enhances the bactericidal activities of neutrophils and macrophages, such as phagocytosis, degranulation, and the formation of NETs, while simultaneously reinforcing mucosal barriers through increased production of protective immunoglobulins and antimicrobial peptides (64, 73, 74). However, in the presence of additional pro-inflammatory signals such as IL-23 and TNF-α, dysregulated or excessive IL-17 can potentiate persistent inflammation and tissue damage. Thus, understanding the intricate balance among these cytokines is crucial to fully appreciating the protective functions of IL-17 in each specific pathological condition, while also acknowledging its potential to become pathogenic if left unchecked.

## 5 Pathological roles of IL-17

Although IL-17 is crucial for immune defense, its overproduction can lead to tissue damage and persistent inflammation (77). In periodontitis, the interaction of IL-17 with IL-1 and tumor necrosis factor (TNF)-α enhances fibroblast secretion of matrix metalloproteinases (MMPs) (78), including MMP-1 and MMP-3 (79), accelerating the breakdown of extracellular matrix proteins and intensifying tissue damage (78). Defective neutrophil recruitment in leukocyte adhesion deficiency type I patients results in excessive production of IL-17 in the periodontal tissue and inflammatory periodontal bone loss (77).

Myeloperoxidase and NETs generated from IL-17-activated neutrophil degranulation promote reactive oxygen species (ROS), causing further tissue damage (11). Additionally, the continuous presence of IL-17 induces various inflammatory mediators in endothelial cells, epithelial cells, and fibroblasts, such as IL-1β, TNF-α, and IL-6, which contribute to the activation of osteoclasts in periodontitis (80).

IL-17 participates in many pathological processes, particularly by inducing autoimmune responses. In rheumatoid arthritis (RA), it promotes bone resorption by inducing the secretion of receptor activator of nuclear factor kappa-B ligand (RANKL) in osteoblasts (81). In ankylosing spondylitis, IL-17 activates the osteoblasts, resulting in osteogenesis and abnormal bone formation (82). Blocking the IL-17 signaling pathway has proven effective in alleviating inflammation in autoimmune conditions (83). Similarly, in periodontitis, numerous studies have shown a strong correlation between the severity of alveolar bone resorption and IL-17 expression levels in gingival tissues (84–86).

**Table 1** provides an overview of IL-17’s dual roles. A key factor driving IL-17’s dual nature is the presence or absence of additional inflammatory modulators and the stage of the disease. In the context of tissue damage and chronic inflammation, IL-17 is often accompanied by other key cytokines like IL-1β and IL-23, which can shift the immune response toward a pathogenic Th17 profile if overproduced. This shift can lead to excessive osteoclast activity and tissue destruction, contributing to bone-related autoimmune and inflammatory diseases such as RA (81), ankylosing spondylitis (82), and periodontitis (78). Conversely, in controlled or resolving inflammation, IL-17 may transiently bolster local defense without escalating to a level that causes irreversible tissue damage. These variations underscore why the same cytokine, IL-17, can be crucial for microbial clearance in one context while exacerbating tissue destruction in another. Notably, the mechanism by which IL-17 contributes to periodontitis appears to be more complex, which is further discussed in subsequent sections.

**Table 1:** Dual roles on the effects of IL-17.

	Role	Function	Refs.
Immune microenvironment under bacterial accumulation	Protection	Enhances neutrophil chemotaxis and activation	(64)
		Promotes antimicrobial peptides	(64)
		NETs release for pathogen clearance	(65–67)
		Boosts mucosal immunity in acute bacterial stimulation	(70–76)
	Pathology	Drives chronic inflammation, and amplifies ROS production via neutrophil degranulation	(11)
		Over-stimulates inflammatory cascades, increasing MMP secretion and extracellular matrix proteins breakdown	(78, 79)
Bone remodeling	Protection	Support bone regeneration when properly regulated	(95, 98, 99, 103)
		Protect alveolar bone by helping clear infections promptly	(68, 69)
	Pathology	Abnormal bone formation in ankylosing spondylitis	(82)
		Excessive IL-17 triggers osteoclastogenesis and bone resorption	(77, 78, 81)

## 6 The role of IL-17 in bone remodeling

Although it is increasingly clear that IL-17’s impact on bone homeostasis depends on its specific cellular targets, the stage of bone remodeling, and local inflammatory signals, the regulatory effects of IL-17 blockage on bone remodeling remain elusive and unpredictable (8). IL-17 can act in concert with IL-23 (the IL-23/IL-17 axis) to drive Th17-mediated bone destruction in autoimmune settings. However, in bacteria-induced contexts that require strong neutrophilic responses, IL-17 can safeguard the bone by preventing excessive bacterial proliferation. In addition, during osteoporosis and bone fracture healing, evidence suggests that IL-17 can assist with bone formation. This section summarizes IL-17’s dual effects on infection-induced bone destruction, its complex roles in experimental arthritis and osteoporosis, and its impact on bone fracture healing in IL-17 knockout mouse models (68, 69, 87–99), as listed in **Table 2**.

**Table 2:** Studies on the effects of IL-17 in various bone-related diseases.

Disease	Intervention	Animal Model	Effect of IL-17 Deficiency on Bone	Key Finding	Refs.
Periodontitis	Oral gavage of *P. gingivalis* for 6 weeks	Female C57BL/6J IL-17RA KO mice	Increased bone resorption	Defective neutrophil migration, reduced expression of LIX and Groα, and impaired chemokine signaling in KO mice	(68)
	Oral gavage of *P. gingivalis* for 6 weeks	Female BALB/c IL-17RA KO mice	Increased bone resorption	Defective neutrophil recruitment, reduced expression of MIP2 and Groα in female KO mice; male KO mice were resistant.	(69)
	Ligature around maxillary molar and flushing for 4 weeks	C57BL/6 *Il17a* ^−/−^ *Il17f* ^−/−^ mice	Increased bone resorption	Slightly higher RANKL/OPG ratio in KO mice, but no significant differences in osteoclast activity.	(15)
	Ligature around maxillary molar for 10 days	C57BL/6 *Il17a* ^−/−^ *Il17f* ^−/−^ mice	Decreased bone resorption	Increased osteoclast activity, and increased radio of *γ-proteobacteria* colonization in KO mice.	(87)
Periapical Periodontitis	Tooth pulp exposed and infected with *P. intermedia*, *S. intermedius*, *F. nucleatum*, and *P. micros*	C57BL/6 IL-17RA KO mice	Increased bone resorption	Increased number of TRAP(+) osteoclasts, increased number of F40/80(+) macrophages, increased number of CD20(+) B cells and increased expression of IL-1α, IL-1β and MIP2 in KO mice.	(88)
	Tooth pulp exposed and infected with *P. intermedia* and *P. gingivalis* for 3 weeks	C57BL/6 IL-17A^−/−^ mice	Decreased bone resorption	IL-17A KO mice did not develop lesions.	(89)
Spondyloarthritis	Serum transfer-induced arthritis	C57BL/6J IL-17A KO mice	Increased bone regeneration	Increased periosteal bone formation by modulating Wnt signaling and sFRP3 in KO mice; increased calvarial osteoblast differentiation and the expression of DKK1.	(90)
Arthritis with Periodontitis	Arthritis induced with *M. tuberculosis* and periodontitis induced with *P. gingivalis*	C57BL/6 IL-17RA KO mice	Decreased bone resorption	Decreased arthritis aggravation and TNF and IL-17 production in KO mice with periodontitis.	(91)
Proteoglycan-Induced Arthritis	Proteoglycan immunization	BALB/c IL-17 KO mice	No significant difference	Decreased expression of RANKL and IL-6, increased number of T cells and the expression of IL-1β in KO mice; IL-17 deficiency did not affect arthritis severity.	(92)
Collagen-Induced Arthritis	Collagen-induced arthritis with allogeneic bone marrow transplantation	C57BL/6 DBA/1J IL-17^−/−^ mice	Decreased bone resorption	Reduced joint inflammation and destruction with a reciprocal regulation of Th17 and Treg populations, reduced the expression of TNF-α, IL-1β, and IL-6 in KO mice.	(93)
Osteoporosis	/	C57BL/6 *LysM*-Cre; IL-17ra^F/F^ mice	Decreased bone resorption	Reduced osteoclast precursor activity and expression of serum CTX; increased the ratio of cortical to trabecular bone in KO mice.	(94)
Post-Menopausal Osteoporosis	Ovariectomy	Female C57BL/6 IL-17RA KO mice	Increased bone resorption	Increased leptin levels, and enhanced adipogenesis in KO mice.	(103)
	Ovariectomy	C57BL/6 IL-17A KO mice	Increased bone resorption	Increased cortical bone loss, reduced bone turnover, and increased bone marrow fat; decreased expression of CTX-I and PINP in KO mice.	(95)
COPD-Induced Osteoporosis	Cigarette smoke exposure	C57BL/6 IL-17A^−/−^ mice	Decreased bone resorption	Reduced osteoclast numbers, lower RANKL expression, and decreased expression of IL-1β and IL-6, protecting against emphysema in KO mice.	(96)
PTH-Induced Osteoporosis	Continuous PTH infusion	C57BL/6 DMP1-8kb–Cre; *Il17ra* ^F/F^ mice	Decreased bone resorption	Decreased RANKL production and osteocytic activity in KO mice.	(97)
Bone Fracture	Drill-hole injury in femur	C57BL/6 *Il17a* ^−/−^ *Il17f* ^−/−^ mice	Decreased bone regeneration	IL-17A produced by Vγ6(+) γδ T cells promoted bone repair by enhancing progenitor cell proliferation and osteoblast differentiation with increased expression of *Alpl* and *Col1a1.*	(98)
	Femur fracture using blunt guillotine	C57BL/6 *Prx1*-cre; *Il17ra* ^F/F^ mice	Decreased bone regeneration	IL-17RA signaling regulated cartilage and bone composition during early fracture healing with increased expression of *Runx2*, *Osterix*, *Bglap* and *Col1a1*.	(99)

### 6.1 Bacteria-induced bone resorption in periodontitis and periapical periodontitis

**Figure 2:**
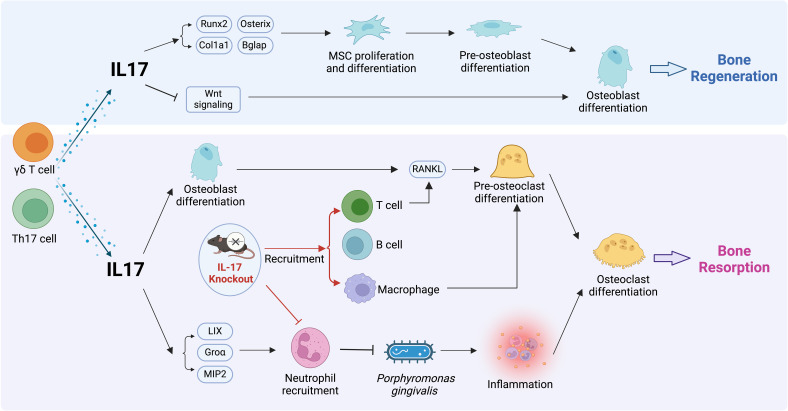
Effects of IL-17 on bone regeneration and resorption. The top panel shows IL-17 promoting mesenchymal stem cell proliferation and osteoblast differentiation via enhanced expression of osteogenic markers and activation of Wnt signaling, leading to bone regeneration. In the lower panel, IL-17 produced by γδ T cells and Th17 cells drives the recruitment of neutrophils, facilitating RANKL-mediated pre-osteoclast transition to osteoclasts and subsequent bone resorption. IL-17-induced chemokines promote neutrophil infiltration and promoting bacterial killing. Studies using knockout mice show IL-17’s impact on the recruitment of other immune cells.

Experimental mouse models for pathogen-induced bone loss are commonly designed to mimic periodontitis or periapical periodontitis. The primary bacterium utilized for inducing periodontitis in experimental models is *P. gingivalis*. Yu et al. first adopted an oral gavage methodology employing *P. gingivalis* in the IL-17RA mouse to establish a periodontitis model. The findings found that IL-17-deficient mice showed increased alveolar bone resorption, thereby highlighting the protective role of IL-17 in preserving bone integrity against *P. gingivalis* (68). The protocol commenced with the administration of antibiotics to suppress the native oral microbiota. Subsequently, *P. gingivalis* was introduced every three days for six weeks. The researchers proposed that this increased susceptibility to *P. gingivalis* in IL-17-deficient mice was attributable to impaired neutrophil recruitment, which led to a compensatory increase in lymphocyte activity and enhancement of RANKL expression and osteoclastogenesis in activated T and B cells (68) (**Figure 2**). Interestingly, this protective role of IL-17 in preserving alveolar bone integrity against *P. gingivalis* appears to exhibit a markedly higher vulnerability in female mice, which may be attributed to sex-specific variations in neutrophil chemokine production (69). Similarly, a recent study shows the protective effect of IL-17 in the late stage of ligature-induced periodontitis (15). The periodontitis model was induced with silk ligature tied on upper second molar in *Il17a* ^−/−^ *Il17f* ^−/−^ mice for four weeks. The study found that the protective effect of IL-17 in the later stages does not depend on γδ T cells (15). However, another study highlighted the role of IL-17 in promoting bone resorption in the early stage of periodontitis. Here, periodontitis induced solely by a silk ligature in *Il17a* ^−/−^ *Il17f* ^−/−^ mice, without any external bacterial stimulation, showed significant alveolar bone resorption after ten days (87). This discrepancy could be due to the method of inducing periodontitis differs, as the accumulation of natural oral bacteria due to the silk ligature differs from the external stimulation by *P. gingivalis*, possibly leading to different roles for IL-17 in periodontitis (100). In the periapical model, contradictory experimental results were observed when different bacterial communities were used to infect the exposed dental pulp (88, 89). Another factor could be the difference in the timing of stimulation, where early-stage inflammation might promote bone resorption mediated by IL-17. A study using *Il17a* ^−/−^ *Il17f* ^−/−^ mice with only silk ligature-induced periodontitis for four weeks showed similar bone resorption in both knockout and wild-type mice by this time point (15), suggesting that IL-17 gradually exhibits a protective role during the later stages of inflammation, potentially due to differences in neutrophil recruitment (68).

### 6.2 Inflammatory bone resorption in arthritis

While IL-17 can be both protective and pathogenic in bacteria-induced periodontitis and periapical periodontitis, it primarily exhibits a pathogenic role in experimental arthritis by exacerbating inflammation and driving pathological bone remodeling (90, 92, 93). On one hand, the deficiency of IL-17 can inhibit the production of pro-inflammatory cytokines such as IL-1β, IL-6, and TNF-α both *in vitro* and *in vivo*. This inhibition may be due to altered cytokine profiles following increased recruitment and proliferation of Treg cells resulting from the IL-17 deficiency (93). Treg cells play a crucial role in preventing autoimmunity and can regulate the severity of collagen-induced arthritis (101). On the other hand, IL-17 upregulates the expression of tartrate-resistant acid phosphatase, MMP9, integrin-β3, Nuclear factor of activated T-cells, cytoplasmic 1, and cathepsin K, which enhances osteoclastogenesis and bone resorption in joints (93). Similarly, periodontitis induced by *P. gingivalis* exacerbates the expression of IL-17 in joint tissues during Mycobacterium tuberculosis-induced arthritis, with a notable reduction in joint deterioration observed after knocking out IL-17RA (92).

Interestingly, studies have found that the absence of IL-17 expression can decrease RANKL expression, yet this has minimal effect on proteoglycan-induced arthritis, with bone resorption levels in IL-17-deficient mice comparable to those in wild-type mice. Furthermore, dominant inflammatory responses led by IFN-γ, which elevate TNF, IL-1β, and IL-6, can induce bone erosion independently of IL-17 (92). Another study highlighted that in K/BxN serum-transferred arthritis, the absence of IL-17 did not affect inflammation or bone resorption under arthritic conditions, but significantly reduced bone regeneration. This effect is potentially due to IL-17 promoting the expression of Wnt antagonists, thereby inhibiting osteoblast differentiation and function (90). The impact of IL-17A on osteoblast differentiation may vary depending on the type of precursor cells, the differentiation state of the osteoblasts, and the duration and timing of exposure (90). IL-17A negatively affects osteoblastogenesis in cultured cells derived from neonatal mouse cranial bones (102).

### 6.3 Bone regeneration in fracture healing

Beyond its role in bone resorption in inflammatory diseases, IL-17 also contributes to bone regeneration and repair during fracture healing by modulating the recruitment, proliferation, and differentiation of mesenchymal progenitor cells (98, 99). Research has shown that high levels of IL-17 are expressed in γδ T cells following bone injury. In *Il17a* ^−/−^ mice, the differentiation of mesenchymal progenitor cells into osteoblasts is significantly reduced, leading to impaired bone regeneration (98). Additionally, studies involving conditional knockout of *Il17ra* in mesenchymal cells of mice have observed delayed secondary fracture healing during the early stages of endochondral ossification. This delay may be caused by reduced migration and proliferation of mesenchymal progenitor cells at the fracture site (99).

### 6.4 Effects on osteoporosis in specific contexts

In different conditions, IL-17 appears to have varying effects on osteoporosis. In postmenopausal osteoporosis, IL-17 knockout seems to promote cortical bone loss and adipogenesis, which are associated with increased production of leptin (95, 103). Interestingly, studies have found that in osteoporosis induced by estrogen deficiency, there is no significant change in the expression of Th effector cytokines in the serum, while the IL-17 signaling pathway can inhibit adipogenesis and the expression of leptin. Leptin can indirectly reduce the ratio of cortical to trabecular bone via the hypothalamus, and can also directly stimulate osteoblasts to promote bone formation (103). Another study using the same model confirmed that while IL-17A deficiency can protect against cortical bone loss, it does not prevent trabecular bone loss. Additionally, IL-17A deficiency inhibits increased expression of CTX-I and PINP, thereby reducing total bone turnover in mice after estrogen deficiency (95).

Conversely, in osteoporosis induced by chronic obstructive pulmonary disease (COPD) and parathyroid hormone (PTH), a deficiency in IL-17A reduces bone loss by decreasing osteoclast activity and RANKL expression (96, 97). Studies have shown that under long-term exposure to cigarette smoke, mice lacking IL-17A exhibit lower levels of IL-1β and IL-6 in the blood, which plays a pivotal role in the inflammatory cascade, potentially indirectly inhibiting osteoclast generation (96). In a model of hyperparathyroidism characterized by persistently elevated PTH, naïve CD4+ cells differentiate into Th17 cells rather than Tregs. Knockout of IL-17RA in mouse osteoblasts inhibits the expression of RANKL induced by PTH, thereby suppressing osteoclast activity (97).

## 7 Controversies surrounding IL-17 in periodontitis

The role of IL-17 in periodontitis remains controversial due to its dual effects on bone homeostasis (104). One important consideration is that IL-17 does not act in isolation. Its interplay with IL-1, IL-6, IL-23, TNF-α, and other local signaling pathways can either drive a protective response that limits bacterial invasion or a pathological response that amplifies inflammation and promotes bone loss (93). Moreover, the timing of IL-17 expression, whether in an early acute inflammatory phase or a more chronic phase, can drastically alter the outcome. For instance, early stages may show predominant osteoclast activation, enhancing bone resorption, whereas later stages might recruit protective neutrophils, thereby reducing stagnant bacterial colonization (15, 68, 87).

Additionally, sex-biased differences in neutrophil chemokine production and T cell responses may lead to distinct IL-17–mediated protective or pathogenic outcomes (69). Environment such as microbial community composition and chronic stimulation, along with host factors like genetic predisposition and hormonal influences, further shape whether IL-17 helps the host defend against pathogens or contributes to tissue damage. Thus, despite being the same cytokine, IL-17’s activity is dictated by the surrounding cellular environment and disease context, illustrating the complexity of therapeutically targeting IL-17 in periodontitis.

Current investigations into the *in vivo* mechanisms of IL-17 primarily utilize experimental murine models of periodontitis to simulate human conditions. Ligature-induced periodontitis is considered a crucial method for studying both innate and acquired immune responses to microbial infections (105). *P. gingivalis* is a common pathogen in human periodontitis; however, many models of periodontitis rely solely on the administration of *P. gingivalis* through oral gavage, which may not accurately replicate the sustained microbial stimulation in the gingival sulcus. Therefore, variations in the induction methods of periodontitis models may lead to inconsistent experimental results, altering the perceived role of IL-17 in periodontitis. Since *P. gingivalis* does not naturally proliferate in the murine oral cavity, to mimic the real oral environment in humans and ensure stable accumulation of the microbiota in the gingival sulcus, the *P. gingivalis*-soaked ligature-induced murine model has been introduced. This model exhibits more severe bone resorption than the simple ligature-induced model (100) and could be considered for future research. Current experimental results suggest that IL-17 may promote osteoclast activity in the early stages of infection but may have a bone-protective role in later stages (15). On one hand, IL-17 deficiency leads to reduced neutrophil recruitment and compensatory lymphocyte enhancement, potentially increasing osteoclast activity and ultimately leading to increased bone resorption (68). On the other hand, as microbial communities vary across different stages (19), IL-17’s protective role for the host may become increasingly important.

The dual roles of IL-17 in bone remodeling present both challenges and opportunities for therapeutic intervention (8). Targeting IL-17 signaling may be beneficial in conditions characterized by excessive bone resorption (46). Monoclonal antibodies against IL-17A or IL-17RA have shown promise in reducing inflammation and bone loss in various autoimmune diseases, such as psoriatic arthritis (106), ankylosing spondylitis (107) and rheumatoid arthritis (108). Furthermore, studies have demonstrated a positive correlation between probing depth, clinical attachment loss, and IL-17A expression levels in the gingiva of patients with chronic periodontitis (109). Some *in vivo* investigations also indicate that IL-17 promotes pathological bone loss in experimental periodontitis (87, 89), and some research validated the therapeutic efficacy of IL-17A antibodies in experimental periodontitis (14). However, IL-17 is essential for controlling *P. gingivalis*-induced infections and preventing excessive microbial proliferation, its overexpression can exacerbate inflammation and bone resorption (68, 69, 88). Therefore, given its essential role in preventing bacterial infections and promoting bone regeneration in fracture healing, caution must be exercised to avoid unintended consequences when using IL-17 blockade treatments. Indeed, IL-17 blockade treatments still require further experimental validation of their safety before clinical application. Of note, other studies have found that, despite the elevated IL-17A levels in periodontal tissues of patients with periodontitis, plasma IL-17A levels may actually be reduced. This reduction may be due to elevated levels of soluble IL-17RA in the plasma, which exerts a neutralizing effect on IL-17A (52, 109). This discrepancy suggests that local pharmacological interventions could be necessary when considering IL-17 blockade treatment for periodontitis. Looking ahead, it is essential for research to delineate IL-17’s specific contributions to periodontitis through the use of refined animal models that more accurately mimic the human oral pathological environment across various stages of disease.

Additionally, future research in periodontitis should focus on the investigation of specific cellular mechanisms and further develop targeted therapies that enable selective inhibition of IL-17 in specific cell types or contexts, such as osteoclast precursors or the early stages of the disease, thereby helping to mitigate bone loss without impairing bone regeneration or protection. Moreover, sex-based differences in IL-17 signaling should be considered to optimize therapeutic strategies and minimize adverse effects.

## 8 Conclusion

In this paper, we review IL-17 acts as a pivotal mediator in immune responses, exhibiting a dual nature that is particularly evident in the context of periodontitis and other bone-related inflammatory diseases. IL-17 operates within a delicate balance, toggling between beneficial and detrimental roles. From a protective standpoint, IL-17 facilitates the recruitment of neutrophils, enhances bacterial killing capabilities, bolsters mucosal immunity and bone regeneration, all of which are crucial for combating bacterial invasions and safeguarding tissues. Conversely, an overactivation of IL-17 can lead to persistent inflammation, tissue damage, and bone loss in periodontitis and other bone-related inflammatory disorders. Whether IL-17 ultimately protects, or harms host tissues depends on local immune regulators, disease stage, and synergistic cytokine networks. This review offers new perspectives on leveraging IL-17 modulation for disease management under conditions such as infection. Future comprehensive studies should focus on developing more physiologically relevant models to clarify IL-17-mediated signaling pathway in specific cells types at different stages of human chronic periodontitis, ultimately aiming to harness its protective effects while mitigating its harmful impacts.

## References

[1] Teles R Benecha HK Preisser JS Moss K Starr JR Corby P. Modelling changes in clinical attachment loss to classify periodontal disease progression. J Clin Periodontol. (2016) 43:426–34. doi: 10.1111/jcpe.12539 PMC5021116 26935472

[2] Teles R Moss K Preisser JS Genco R Giannobile WV Corby P. Patterns of periodontal disease progression based on linear mixed models of clinical attachment loss. J Clin Periodontol. (2018) 45:15–25. doi: 10.1111/jcpe.12827 28985450

[3] Contreras A Herrera JA Soto JE Arce RM Jaramillo A Botero JE. Periodontitis is associated with preeclampsia in pregnant women. J Periodontol. (2006) 77:182–8. doi: 10.1902/jop.2006.050020 16460242

[4] Dommisch H Kuzmanova D Jönsson D Grant M Chapple I. Effect of micronutrient malnutrition on periodontal disease and periodontal therapy. Periodontol 2000. (2018) 78:129–53. doi: 10.1111/prd.12233 30198127

[5] Genco RJ Borgnakke WS. Diabetes as a potential risk for periodontitis: association studies. Periodontol 2000. (2020) 83:40–5. doi: 10.1111/prd.12270 32385881

[6] Genco RJ Sanz M. Clinical and public health implications of periodontal and systemic diseases: an overview. Periodontol 2000. (2020) 83:7–13. doi: 10.1111/prd.12344 32385880

[7] Sabbah W Gomaa N Gireesh A. Stress, allostatic load, and periodontal diseases. Periodontol 2000. (2018) 78:154–61. doi: 10.1111/prd.12238 30198126

[8] Mills KHG. Il-17 and il-17-producing cells in protection versus pathology. Nat Rev Immunol. (2023) 23:38–54. doi: 10.1038/s41577-022-00746-9 35790881 PMC9255545

[9] Veldhoen M. Interleukin 17 is a chief orchestrator of immunity. Nat Immunol. (2017) 18:612–21. doi: 10.1038/ni.3742 28518156

[10] Dubois V Chatagnon J Thiriard A Bauderlique-Le-Roy H Debrie AS Coutte L. Suppression of mucosal th17 memory responses by acellular pertussis vaccines enhances nasal bordetella pertussis carriage. NPJ Vaccines. (2021) 6:6. doi: 10.1038/s41541-020-00270-8 33420041 PMC7794405

[11] Fan X Shu P Wang Y Ji N Zhang D. Interactions between neutrophils and T-helper 17 cells. Front Immunol. (2023) 14:1279837. doi: 10.3389/fimmu.2023.1279837 37920459 PMC10619153

[12] Kim TS Silva LM Theofilou VI Greenwell-Wild T Li L Williams DW. Neutrophil extracellular traps and extracellular histones potentiate il-17 inflammation in periodontitis. J Exp Med. (2023) 220:e20221751. doi: 10.1084/jem.20221751 37261457 PMC10236943

[13] Wang Y Xue N Wang Z Zeng X Ji N Chen Q. Targeting th17 cells: A promising strategy to treat oral mucosal inflammatory diseases. Front Immunol. (2023) 14:1236856. doi: 10.3389/fimmu.2023.1236856 37564654 PMC10410157

[14] Pacheco CMF Maltos KLM Shehabeldin MS Thomas LL Zhuang Z Yoshizawa S. Local sustained delivery of anti-il-17a antibodies limits inflammatory bone loss in murine experimental periodontitis. J Immunol. (2021) 206:2386–92. doi: 10.4049/jimmunol.2001432 PMC10415091 33952619

[15] Wilharm A Binz C Sandrock I Rampoldi F Lienenklaus S Blank E. Interleukin-17 is disease promoting in early stages and protective in late stages of experimental periodontitis. PloS One. (2022) 17:e0265486. doi: 10.1371/journal.pone.0265486 35298525 PMC8929577

[16] Bartold PM. Lifestyle and periodontitis: the emergence of personalized periodontics. Periodontol 2000. (2018) 78:7–11. doi: 10.1111/prd.12237 30198129

[17] Hajishengallis G Lamont RJ. Dancing with the stars: how choreographed bacterial interactions dictate nososymbiocity and give rise to keystone pathogens, accessory pathogens, and pathobionts. Trends Microbiol. (2016) 24:477–89. doi: 10.1016/j.tim.2016.02.010 PMC4874887 26968354

[18] Abusleme L Hoare A Hong BY Diaz PI. Microbial signatures of health, gingivitis, and periodontitis. Periodontol 2000. (2021) 86:57–78. doi: 10.1111/prd.12362 33690899

[19] Wade WG. Has the use of molecular methods for the characterization of the human oral microbiome changed our understanding of the role of bacteria in the pathogenesis of periodontal disease? J Clin Periodontol. (2011) 38:7–16. doi: 10.1111/j.1600-051X.2010.01679.x 21323699

[20] Socransky SS Haffajee AD Cugini MA Smith C Kent RL Jr. Microbial complexes in subgingival plaque. J Clin Periodontol. (1998) 25:134–44. doi: 10.1111/j.1600-051X.1998.tb02419.x 9495612

[21] Darveau RP Hajishengallis G Curtis MA. Porphyromonas gingivalis as a potential community activist for disease. J Dental Res. (2012) 91:816–20. doi: 10.1177/0022034512453589 PMC3420389 22772362

[22] Hajishengallis G Lambris JD. Microbial manipulation of receptor crosstalk in innate immunity. Nat Rev Immunol. (2011) 11:187–200. doi: 10.1038/nri2918 21350579 PMC3077082

[23] Barth K Remick DG Genco CA. Disruption of immune regulation by microbial pathogens and resulting chronic inflammation. J Cell Physiol. (2013) 228:1413–22. doi: 10.1002/jcp.24299 PMC3995356 23255141

[24] Hajishengallis G Liang S Payne Mark A Hashim A Jotwani R Eskan Mehmet A. Low-abundance biofilm species orchestrates inflammatory periodontal disease through the commensal microbiota and complement. Cell Host Microbe. (2011) 10:497–506. doi: 10.1016/j.chom.2011.10.006 22036469 PMC3221781

[25] Moutsopoulos NM Kling HM Angelov N Jin W Palmer RJ Nares S. Porphyromonas gingivalis promotes th17 inducing pathways in chronic periodontitis. J Autoimmun. (2012) 39:294–303. doi: 10.1016/j.jaut.2012.03.003 22560973 PMC3416947

[26] Gaddis DE Maynard CL Weaver CT Michalek SM Katz J. Role of tlr2-dependent il-10 production in the inhibition of the initial ifn-Γ T cell response to porphyromonas gingivalis. J Leukocyte Biol. (2013) 93:21–31. doi: 10.1189/jlb.0512220 23077245 PMC3525832

[27] Yoshioka H Yoshimura A Kaneko T Golenbock DT Hara Y. Analysis of the activity to induce toll-like receptor (Tlr)2- and tlr4-mediated stimulation of supragingival plaque. J Periodontol. (2008) 79:920–8. doi: 10.1902/jop.2008.070516 18454672

[28] Hajishengallis G Darveau RP Curtis MA. The keystone-pathogen hypothesis. Nat Rev Microbiol. (2012) 10:717–25. doi: 10.1038/nrmicro2873 PMC3498498 22941505

[29] Stabholz A Soskolne WA Shapira L. Genetic and environmental risk factors for chronic periodontitis and aggressive periodontitis. Periodontol 2000. (2010) 53:138–53. doi: 10.1111/j.1600-0757.2010.00340.x 20403110

[30] Zhou Q Leeman SE Amar S. Signaling mechanisms in the restoration of impaired immune function due to diet-induced obesity. Proc Natl Acad Sci. (2011) 108:2867–72. doi: 10.1073/pnas.1019270108 PMC3041076 21282635

[31] Laine ML Crielaard W Loos BG. Genetic susceptibility to periodontitis. Periodontol 2000. (2012) 58:37–68. doi: 10.1111/j.1600-0757.2011.00415.x 22133366

[32] Divaris K Monda KL North KE Olshan AF Reynolds LM Hsueh W-C. Exploring the genetic basis of chronic periodontitis: A genome-wide association study. Hum Mol Genet. (2013) 22:2312–24. doi: 10.1093/hmg/ddt065 PMC3652417 23459936

[33] Lindroth AM Park YJ. Epigenetic biomarkers: A step forward for understanding periodontitis. J Periodontal Implant Sci. (2013) 43:111–20. doi: 10.5051/jpis.2013.43.3.111 PMC3701832 23837125

[34] Hajishengallis G. Too old to fight? Aging and its toll on innate immunity. Mol Oral Microbiol. (2010) 25:25–37. doi: 10.1111/j.2041-1014.2009.00562.x 20305805 PMC2839454

[35] Eskan MA Jotwani R Abe T Chmelar J Lim J-H Liang S. The leukocyte integrin antagonist del-1 inhibits il-17-mediated inflammatory bone loss. Nat Immunol. (2012) 13:465–73. doi: 10.1038/ni.2260 PMC3330141 22447028

[36] Kurgan S Kantarci A. Molecular basis for immunohistochemical and inflammatory changes during progression of gingivitis to periodontitis. Periodontol 2000. (2018) 76:51–67. doi: 10.1111/prd.12146 29194785

[37] Bosshardt DD Lang NP. The junctional epithelium: from health to disease. J Dental Res. (2005) 84:9–20. doi: 10.1177/154405910508400102 15615869

[38] Nussbaum G Shapira L. How has neutrophil research improved our understanding of periodontal pathogenesis? J Clin Periodontol. (2011) 38:49–59. doi: 10.1111/j.1600-051X.2010.01678.x 21323704

[39] Vilotić A Nacka-Aleksić M Pirković A Bojić-Trbojević Ž Dekanski D Jovanović Krivokuća M. Il-6 and il-8: an overview of their roles in healthy and pathological pregnancies. Int J Mol Sci. (2022) 23:14574. doi: 10.3390/ijms232314574 36498901 PMC9738067

[40] Sharygin D Koniaris LG Wells C Zimmers TA Hamidi T. Role of cd14 in human disease. Immunology. (2023) 169:260–70. doi: 10.1111/imm.13634 PMC10591340 36840585

[41] Stevens AJ Harris AR Gerdts J Kim KH Trentesaux C Ramirez JT. Programming multicellular assembly with synthetic cell adhesion molecules. Nature. (2023) 614:144–52. doi: 10.1038/s41586-022-05622-z PMC9892004 36509107

[42] Zhai YJ Feng Y Ma X Ma F. Defensins: defenders of human reproductive health. Hum Reprod Update. (2023) 29:126–54. doi: 10.1093/humupd/dmac032 PMC9825273 36130055

[43] Kawai T Ikegawa M Ori D Akira S. Decoding toll-like receptors: recent insights and perspectives in innate immunity. Immunity. (2024) 57:649–73. doi: 10.1016/j.immuni.2024.03.004 38599164

[44] Zhang J Huang S Zhu Z Gatt A Liu J. E-selectin in vascular pathophysiology. Front Immunol. (2024) 15:1401399. doi: 10.3389/fimmu.2024.1401399 39100681 PMC11294169

[45] Zhang Q Shen X Yuan X Huang J Zhu Y Zhu T. Lipopolysaccharide binding protein resists hepatic oxidative stress by regulating lipid droplet homeostasis. Nat Commun. (2024) 15:3213. doi: 10.1038/s41467-024-47553-5 38615060 PMC11016120

[46] Huangfu L Li R Huang Y Wang S. The il-17 family in diseases: from bench to bedside. Signal Transduct Target Ther. (2023) 8:402. doi: 10.1038/s41392-023-01620-3 37816755 PMC10564932

[47] Rouvier E Luciani MF Mattéi MG Denizot F Golstein P. Ctla-8, cloned from an activated T cell, bearing au-rich messenger rna instability sequences, and homologous to a herpesvirus saimiri gene. J Immunol. (1993) 150:5445–56. doi: 10.4049/jimmunol.150.12.5445 8390535

[48] Ely LK Fischer S Garcia KC. Structural basis of receptor sharing by interleukin 17 cytokines. Nat Immunol. (2009) 10:1245–51. doi: 10.1038/ni.1813 PMC2783927 19838198

[49] Pappu R Ramirez-Carrozzi V Sambandam A. The interleukin-17 cytokine family: critical players in host defence and inflammatory diseases. Immunology. (2011) 134:8–16. doi: 10.1111/j.1365-2567.2011.03465.x 21726218 PMC3173690

[50] Langrish CL Chen Y Blumenschein WM Mattson J Basham B Sedgwick JD. Il-23 drives a pathogenic T cell population that induces autoimmune inflammation. J Exp Med. (2005) 201:233–40. doi: 10.1084/jem.20041257 PMC2212798 15657292

[51] Sohda M Misumi Y Tashiro K Yamazaki M Saku T Oda K. Identification of a soluble isoform of human il-17ra generated by alternative splicing. Cytokine. (2013) 64:642–5. doi: 10.1016/j.cyto.2013.09.012 24084331

[52] Rodríguez-Montaño R Bernard-Medina AG Oregon-Romero E Martínez-Rodríguez V Pita-López ML Gómez-Meda BC. Il-23/il-17 axis and soluble receptors isoforms sil-23r and sil-17ra in patients with rheumatoid arthritis-presenting periodontitis. J Clin Lab Anal. (2021) 35:e23963. doi: 10.1002/jcla.23963 34403509 PMC8418468

[53] Ruterbusch M Pruner KB Shehata L Pepper M. *In vivo* cd4(+) T cell differentiation and function: revisiting the th1/th2 paradigm. Annu Rev Immunol. (2020) 38:705–25. doi: 10.1146/annurev-immunol-103019-085803 32340571

[54] Veldhoen M Hocking RJ Atkins CJ Locksley RM Stockinger B. Tgfbeta in the context of an inflammatory cytokine milieu supports *de novo* differentiation of il-17-producing T cells. Immunity. (2006) 24:179–89. doi: 10.1016/j.immuni.2006.01.001 16473830

[55] Acosta-Rodriguez EV Napolitani G Lanzavecchia A Sallusto F. Interleukins 1beta and 6 but not transforming growth factor-beta are essential for the differentiation of interleukin 17-producing human T helper cells. Nat Immunol. (2007) 8:942–9. doi: 10.1038/ni1496 17676045

[56] Ivanov II McKenzie BS Zhou L Tadokoro CE Lepelley A Lafaille JJ. The orphan nuclear receptor rorgammat directs the differentiation program of proinflammatory il-17+ T helper cells. Cell. (2006) 126:1121–33. doi: 10.1016/j.cell.2006.07.035 16990136

[57] Luo X Chen O Wang Z Bang S Ji J Lee SH. Il-23/il-17a/trpv1 axis produces mechanical pain via macrophage-sensory neuron crosstalk in female mice. Neuron. (2021) 109:2691–706.e5. doi: 10.1016/j.neuron.2021.06.015 34473953 PMC8425601

[58] Schlegel PM Steiert I Kötter I Müller CA. B cells contribute to heterogeneity of il-17 producing cells in rheumatoid arthritis and healthy controls. PloS One. (2013) 8:e82580. doi: 10.1371/journal.pone.0082580 24340045 PMC3855537

[59] Shimura E Shibui A Narushima S Nambu A Yamaguchi S Akitsu A. Potential role of myeloid cell/eosinophil-derived il-17 in lps-induced endotoxin shock. Biochem Biophys Res Commun. (2014) 453:1–6. doi: 10.1016/j.bbrc.2014.09.004 25204502 PMC4250284

[60] Beura LK Hamilton SE Bi K Schenkel JM Odumade OA Casey KA. Normalizing the environment recapitulates adult human immune traits in laboratory mice. Nature. (2016) 532:512–6. doi: 10.1038/nature17655 PMC4871315 27096360

[61] Dutzan N Abusleme L Konkel JE Moutsopoulos NM. Isolation, characterization and functional examination of the gingival immune cell network. J Vis Exp. (2016) 108):53736. doi: 10.3791/53736 PMC4828169 26967370

[62] Dutzan N Konkel JE Greenwell-Wild T Moutsopoulos NM. Characterization of the human immune cell network at the gingival barrier. Mucosal Immunol. (2016) 9:1163–72. doi: 10.1038/mi.2015.136 PMC4820049 26732676

[63] Dutzan N Kajikawa T Abusleme L Greenwell-Wild T Zuazo CE Ikeuchi T. A dysbiotic microbiome triggers T(H)17 cells to mediate oral mucosal immunopathology in mice and humans. Sci Transl Med. (2018) 10:eaat0797. doi: 10.1126/scitranslmed.aat0797 30333238 PMC6330016

[64] Aujla SJ Chan YR Zheng M Fei M Askew DJ Pociask DA. Il-22 mediates mucosal host defense against gram-negative bacterial pneumonia. Nat Med. (2008) 14:275–81. doi: 10.1038/nm1710 PMC2901867 18264110

[65] Burn GL Foti A Marsman G Patel DF Zychlinsky A. The neutrophil. Immunity. (2021) 54:1377–91. doi: 10.1016/j.immuni.2021.06.006 34260886

[66] Fu J Zong X Jin M Min J Wang F Wang Y. Mechanisms and regulation of defensins in host defense. Signal Transduct Target Ther. (2023) 8:300. doi: 10.1038/s41392-023-01553-x 37574471 PMC10423725

[67] Wang H Kim SJ Lei Y Wang S Wang H Huang H. Neutrophil extracellular traps in homeostasis and disease. Signal Transduct Target Ther. (2024) 9:235. doi: 10.1038/s41392-024-01933-x 39300084 PMC11415080

[68] Yu JJ Ruddy MJ Wong GC Sfintescu C Baker PJ Smith JB. An essential role for il-17 in preventing pathogen-initiated bone destruction: recruitment of neutrophils to inflamed bone requires il-17 receptor-dependent signals. Blood. (2007) 109:3794–802. doi: 10.1182/blood-2005-09-010116 PMC1874584 17202320

[69] Yu JJ Ruddy MJ Conti HR Boonanantanasarn K Gaffen SL. The interleukin-17 receptor plays a gender-dependent role in host protection against porphyromonas gingivalis-induced periodontal bone loss. Infect Immun. (2008) 76:4206–13. doi: 10.1128/iai.01209-07 PMC2519446 18591228

[70] Higgins SC Jarnicki AG Lavelle EC Mills KH. Tlr4 mediates vaccine-induced protective cellular immunity to bordetella pertussis: role of il-17-producing T cells. J Immunol. (2006) 177:7980–9. doi: 10.4049/jimmunol.177.11.7980 17114471

[71] Lu YJ Gross J Bogaert D Finn A Bagrade L Zhang Q. Interleukin-17a mediates acquired immunity to pneumococcal colonization. PloS Pathog. (2008) 4:e1000159. doi: 10.1371/journal.ppat.1000159 18802458 PMC2528945

[72] Mues N Martin RJ Alam R Schaunaman N Dimasuay KG Kolakowski C. Bacterial DNA amplifies neutrophilic inflammation in il-17-exposed airways. ERJ Open Res. (2023) 9:00474–2022. doi: 10.1183/23120541.00474-2022 PMC9868970 36699649

[73] Borkner L Curham LM Wilk MM Moran B Mills KHG. Il-17 mediates protective immunity against nasal infection with bordetella pertussis by mobilizing neutrophils, especially siglec-F(+) neutrophils. Mucosal Immunol. (2021) 14:1183–202. doi: 10.1038/s41385-021-00407-5 PMC8379078 33976385

[74] Solans L Debrie AS Borkner L Aguiló N Thiriard A Coutte L. Il-17-dependent siga-mediated protection against nasal bordetella pertussis infection by live attenuated bpze1 vaccine. Mucosal Immunol. (2018) 11:1753–62. doi: 10.1038/s41385-018-0073-9 30115992

[75] Allen AC Wilk MM Misiak A Borkner L Murphy D Mills KHG. Sustained protective immunity against bordetella pertussis nasal colonization by intranasal immunization with a vaccine-adjuvant combination that induces il-17-secreting T(Rm) cells. Mucosal Immunol. (2018) 11:1763–76. doi: 10.1038/s41385-018-0080-x 30127384

[76] Wilk MM Borkner L Misiak A Curham L Allen AC Mills KHG. Immunization with Whole Cell but Not Acellular Pertussis Vaccines Primes Cd4 T(Rm) Cells That Sustain Protective Immunity against Nasal Colonization with Bordetella Pertussis. Emerg Microbes Infect. (2019) 8:169–85. doi: 10.1080/22221751.2018.1564630 PMC6455184 30866771

[77] Moutsopoulos NM Konkel J Sarmadi M Eskan MA Wild T Dutzan N. Defective neutrophil recruitment in leukocyte adhesion deficiency type I disease causes local il-17-driven inflammatory bone loss. Sci Transl Med. (2014) 6:229ra40. doi: 10.1126/scitranslmed.3007696 PMC4090608 24670684

[78] Beklen A Ainola M Hukkanen M Gürgan C Sorsa T Konttinen YT. Mmps, il-1, and tnf are regulated by il-17 in periodontitis. J Dent Res. (2007) 86:347–51. doi: 10.1177/154405910708600409 17384030

[79] Okić Đorđević I Kukolj T Živanović M Momčilović S Obradović H Petrović A. The role of doxycycline and il-17 in regenerative potential of periodontal ligament stem cells: implications in periodontitis. Biomolecules. (2023) 13:1437. doi: 10.3390/biom13101437 37892119 PMC10604178

[80] Pelletier M Maggi L Micheletti A Lazzeri E Tamassia N Costantini C. Evidence for a cross-talk between human neutrophils and th17 cells. Blood. (2010) 115:335–43. doi: 10.1182/blood-2009-04-216085 19890092

[81] Ju J-H Cho M-L Jhun J-Y Park M-J Oh H-J Min S-Y. Oral administration of type-ii collagen suppresses il-17-associated rankl expression of cd4+ T cells in collagen-induced arthritis. Immunol Lett. (2008) 117:16–25. doi: 10.1016/j.imlet.2007.09.011 18242716

[82] Jo S Wang SE Lee YL Kang S Lee B Han J. Il-17a induces osteoblast differentiation by activating jak2/stat3 in ankylosing spondylitis. Arthritis Res Ther. (2018) 20:115. doi: 10.1186/s13075-018-1582-3 29880011 PMC5992730

[83] Warren RB Blauvelt A Bagel J Papp KA Yamauchi P Armstrong A. Bimekizumab versus adalimumab in plaque psoriasis. N Engl J Med. (2021) 385:130–41. doi: 10.1056/NEJMoa2102388 33891379

[84] Ohyama H Kato-Kogoe N Kuhara A Nishimura F Nakasho K Yamanegi K. The involvement of il-23 and the th17 pathway in periodontitis. J Dent Res. (2009) 88:633–8. doi: 10.1177/0022034509339889 19605880

[85] Allam JP Duan Y Heinemann F Winter J Götz W Deschner J. Il-23-producing cd68 + Macrophage-like cells predominate within an il-17-polarized infiltrate in chronic periodontitis lesions. J Clin Periodontol. (2011) 38:879–86. doi: 10.1111/j.1600-051X.2011.01752.x 21883359

[86] Okui T Aoki Y Ito H Honda T Yamazaki K. The presence of il-17+/foxp3+ Double-positive cells in periodontitis. J Dent Res. (2012) 91:574–9. doi: 10.1177/0022034512446341 22522772

[87] Tsukasaki M Komatsu N Nagashima K Nitta T Pluemsakunthai W Shukunami C. Host defense against oral microbiota by bone-damaging T cells. Nat Commun. (2018) 9:701. doi: 10.1038/s41467-018-03147-6 29453398 PMC5816021

[88] AlShwaimi E Berggreen E Furusho H Rossall JC Dobeck J Yoganathan S. Il-17 receptor a signaling is protective in infection-stimulated periapical bone destruction. J Immunol. (2013) 191:1785–91. doi: 10.4049/jimmunol.1202194 PMC3767040 23863904

[89] Oseko F Yamamoto T Akamatsu Y Kanamura N Iwakura Y Imanishi J. Il-17 is involved in bone resorption in mouse periapical lesions. Microbiol Immunol. (2009) 53:287–94. doi: 10.1111/j.1348-0421.2009.00123.x 19457170

[90] Shaw AT Maeda Y Gravallese EM. Il-17a deficiency promotes periosteal bone formation in a model of inflammatory arthritis. Arthritis Res Ther. (2016) 18:104. doi: 10.1186/s13075-016-0998-x 27165410 PMC4863346

[91] de Aquino SG Talbot J Sônego F Turato WM Grespan R Avila-Campos MJ. The aggravation of arthritis by periodontitis is dependent of il-17 receptor a activation. J Clin Periodontol. (2017) 44:881–91. doi: 10.1111/jcpe.12743 28498497

[92] Doodes PD Cao Y Hamel KM Wang Y Farkas B Iwakura Y. Development of proteoglycan-induced arthritis is independent of il-17. J Immunol. (2008) 181:329–37. doi: 10.4049/jimmunol.181.1.329 PMC2495052 18566398

[93] Park MJ Park HS Oh HJ Lim JY Yoon BY Kim HY. Il-17-deficient allogeneic bone marrow transplantation prevents the induction of collagen-induced arthritis in dba/1j mice. Exp Mol Med. (2012) 44:694–705. doi: 10.3858/emm.2012.44.11.078 23114425 PMC3509186

[94] Roberts JL Mella-Velazquez G Dar HY Liu G Drissi H. Deletion of il-17ra in osteoclast precursors increases bone mass by decreasing osteoclast precursor abundance. Bone. (2022) 157:116310. doi: 10.1016/j.bone.2021.116310 34973492 PMC10084774

[95] Scheffler JM Grahnemo L Engdahl C Drevinge C Gustafsson KL Corciulo C. Interleukin 17a: A janus-faced regulator of osteoporosis. Sci Rep. (2020) 10:5692. doi: 10.1038/s41598-020-62562-2 32231224 PMC7105470

[96] Xiong J Tian J Zhou L Le Y Sun Y. Interleukin-17a deficiency attenuated emphysema and bone loss in mice exposed to cigarette smoke. Int J Chron Obstruct Pulmon Dis. (2020) 15:301–10. doi: 10.2147/copd.S235384 PMC7020917 32103929

[97] Li JY Yu M Tyagi AM Vaccaro C Hsu E Adams J. Il-17 receptor signaling in osteoblasts/osteocytes mediates pth-induced bone loss and enhances osteocytic rankl production. J Bone Miner Res. (2019) 34:349–60. doi: 10.1002/jbmr.3600 30399207

[98] Ono T Okamoto K Nakashima T Nitta T Hori S Iwakura Y. Il-17-producing Γδ T cells enhance bone regeneration. Nat Commun. (2016) 7:10928. doi: 10.1038/ncomms10928 26965320 PMC4792964

[99] Roberts JL Kapfhamer D Devarapalli V Drissi H. Il-17ra signaling in prx1+ Mesenchymal cells influences fracture healing in mice. Int J Mol Sci. (2024) 25:3751. doi: 10.3390/ijms25073751 38612562 PMC11011315

[100] Lin P Niimi H Ohsugi Y Tsuchiya Y Shimohira T Komatsu K. Application of ligature-induced periodontitis in mice to explore the molecular mechanism of periodontal disease. Int J Mol Sci. (2021) 22:8900. doi: 10.3390/ijms22168900 34445604 PMC8396362

[101] Bluestone JA. Regulatory T-cell therapy: is it ready for the clinic? Nat Rev Immunol. (2005) 5:343–9. doi: 10.1038/nri1574 15775994

[102] Kim YG Park JW Lee JM Suh JY Lee JK Chang BS. Il-17 inhibits osteoblast differentiation and bone regeneration in rat. Arch Oral Biol. (2014) 59:897–905. doi: 10.1016/j.archoralbio.2014.05.009 24907519

[103] Goswami J Hernández-Santos N Zuniga LA Gaffen SL. A bone-protective role for il-17 receptor signaling in ovariectomy-induced bone loss. Eur J Immunol. (2009) 39:2831–9. doi: 10.1002/eji.200939670 PMC2811493 19731364

[104] Gravallese EM Schett G. Effects of the il-23-il-17 pathway on bone in spondyloarthritis. Nat Rev Rheumatol. (2018) 14:631–40. doi: 10.1038/s41584-018-0091-8 30266977

[105] de Molon RS de Avila ED Boas Nogueira AV Chaves de Souza JA Avila-Campos MJ de Andrade CR. Evaluation of the host response in various models of induced periodontal disease in mice. J Periodontol. (2014) 85:465–77. doi: 10.1902/jop.2013.130225 23805811

[106] Steel KJA Srenathan U Ridley M Durham LE Wu SY Ryan SE. Polyfunctional, proinflammatory, tissue-resident memory phenotype and function of synovial interleukin-17a+Cd8+ T cells in psoriatic arthritis. Arthritis Rheumatol. (2020) 72:435–47. doi: 10.1002/art.41156 PMC7065207 31677365

[107] Wang R Maksymowych WP. Targeting the interleukin-23/interleukin-17 inflammatory pathway: successes and failures in the treatment of axial spondyloarthritis. Front Immunol. (2021) 12:715510. doi: 10.3389/fimmu.2021.715510 34539646 PMC8446672

[108] Smolen JS Agarwal SK Ilivanova E Xu XL Miao Y Zhuang Y. A randomised phase ii study evaluating the efficacy and safety of subcutaneously administered ustekinumab and guselkumab in patients with active rheumatoid arthritis despite treatment with methotrexate. Ann Rheum Dis. (2017) 76:831–9. doi: 10.1136/annrheumdis-2016-209831 PMC5530337 28087506

[109] Rodríguez-Montaño R Ruiz-Gutiérrez A Martínez-Rodríguez V Gómez-Sandoval JR Guzmán-Flores JM Becerra-Ruiz JS. Levels of il-23/il-17 axis in plasma and gingival tissue of periodontitis patients according to the new classification. Appl Sci. (2022) 12:8051. doi: 10.3390/app12168051

